# Experimental Human Pneumococcal Carriage Augments IL-17A-dependent T-cell Defence of the Lung

**DOI:** 10.1371/journal.ppat.1003274

**Published:** 2013-03-28

**Authors:** Adam K. A. Wright, Mathieu Bangert, Jenna F. Gritzfeld, Daniela M. Ferreira, Kondwani C. Jambo, Angela D. Wright, Andrea M. Collins, Stephen B. Gordon

**Affiliations:** 1 Respiratory Infection Group, Liverpool School of Tropical Medicine, Liverpool, United Kingdom; 2 National Institute for Health Research Biomedical Research Centre in Microbial Diseases, Royal Liverpool and Broadgreen University Hospitals NHS Trust, Liverpool, United Kingdom; 3 Malawi-Liverpool-Wellcome Trust Clinical Research Programme, Chichiri, Blantyre, Malawi; 4 Comprehensive Local Research Network, Royal Liverpool and Broadgreen University Hospital Trust, Liverpool, United Kingdom; The University of Texas Health Science Center at San Antonio, United States of America

## Abstract

Pneumococcal carriage is both immunising and a pre-requisite for mucosal and systemic disease. Murine models of pneumococcal colonisation show that IL-17A-secreting CD4^+^ T-cells (Th-17 cells) are essential for clearance of pneumococci from the nasopharynx. Pneumococcal-responding IL-17A-secreting CD4^+^ T-cells have not been described in the adult human lung and it is unknown whether they can be elicited by carriage and protect the lung from pneumococcal infection. We investigated the direct effect of experimental human pneumococcal nasal carriage (EHPC) on the frequency and phenotype of cognate CD4^+^ T-cells in broncho-alveolar lavage and blood using multi-parameter flow cytometry. We then examined whether they could augment *ex vivo* alveolar macrophage killing of pneumococci using an *in vitro* assay. We showed that human pneumococcal carriage leads to a 17.4-fold (p = 0.007) and 8-fold (p = 0.003) increase in the frequency of cognate IL-17A^+^ CD4^+^ T-cells in BAL and blood, respectively. The phenotype with the largest proportion were TNF^+^/IL-17A^+^ co-producing CD4^+^ memory T-cells (p<0.01); IFNγ^+^ CD4^+^ memory T-cells were not significantly increased following carriage. Pneumococci could stimulate large amounts of IL-17A protein from BAL cells in the absence of carriage but in the presence of cognate CD4^+^ memory T-cells, IL-17A protein levels were increased by a further 50%. Further to this we then show that alveolar macrophages, which express IL-17A receptors A and C, showed enhanced killing of opsonised pneumococci when stimulated with rhIL-17A (p = 0.013). Killing negatively correlated with RC (r = −0.9, p = 0.017) but not RA expression. We conclude that human pneumococcal carriage can increase the proportion of lung IL-17A-secreting CD4^+^ memory T-cells that may enhance innate cellular immunity against pathogenic challenge. These pathways may be utilised to enhance vaccine efficacy to protect the lung against pneumonia.

## Author Summary

Pneumococcal carriage is an important step in the development of cellular and humoral pneumococcal immunity but paradoxically may lead to mucosal diseases such as pneumonia. The frequency of carriage and pneumonia in young healthy adults is very low despite frequent exposures suggesting the presence of appropriate mucosal defences. Lung mucosal immunity against the pneumococcus is poorly described in humans and lags behind recent advances in our understanding of protective cellular responses in mice. We have therefore developed a method to experimentally induce pneumococcal carriage in healthy adults in order to provide a mechanistic insight into the protective responses elicited at the lung surface. We were able to produce carriage in healthy adults and show that – in the absence of respiratory symptoms or local lung inflammation – pneumococcal-responding (adaptive) cellular responses are increased to a large extent. We also provide evidence of cellular cross-talk between lung sentinels and the pneumococcal-responding adaptive response that may help prevent lung infection in humans. Manipulation of this response may provide novel therapeutic approaches to prevent pneumonia. Furthermore these tools allow better interpretation of defective responses in at risk individuals such as the elderly.

## Introduction

Nasopharyngeal colonisation with *Streptococcus pneumoniae* (the pneumococcus) peaks in prevalence at 2–3 years of age [1] and declines thereafter becoming 10% or less in adult-hood and undetectable in the elderly [2]. Perturbations in host defence and/or increased pneumococcal pathogenicity facilitate colonisation and increase the frequency of progression to mucosal diseases such as pneumonia [3]. Pneumonia is the leading cause of hospitalisation of children in the USA [4]. Elderly populations are also highly susceptible to pneumonia [5]. Pneumococcal carriage is critical in transmission and disease but paradoxically it is also essential for the development of adaptive immunity.

Pneumococcal nasopharyngeal colonisation leads to the establishment of antigen specific memory CD4^+^ T-cells [6], [7] and specific antibody [8], [9] at systemic and mucosal sites in mice. It is well established in mice that, in concert with specific antibody and innate immunity, pneumococcal-responding interleukin-17^+^ (IL-17A^+^) and not interferon-gamma^+^ (IFNγ^+^) CD4^+^ T-cells (Th-17 cells) are essential for protection against pneumococcal carriage [6], [7] but their role in the lung is less clear. Pneumococcal lung infection in mice leads to the significant recruitment of CD4^+^ T-cells into the lungs [3], [7], [10], [11]. T cells are associated with protection from pneumococcal pneumonia in some models [3] but not others [8], [12] possibly owing to variation in host genetic background and the murine bacterial challenge model used.

In humans, increased rates of pneumococcal carriage in children [13] and clinical cases of pneumonia in adults [14] were associated with a reduction in circulating Th-1 (IFNγ^+^) CD4^+^ T-cells. Polymorphisms in the IL-17A gene are associated with increased pneumococcal colonisation [15] and lung infection [16]. IL-17A and IFNγ can be detected in pneumococcal stimulated blood samples [17]–[19] and tonsillar mononuclear cells [20]. T cells with a Th-1 [21] and Th-17 [22] phenotype have been described in the human airway but their specificity for pneumococcus has not been shown and it is unknown whether they are directly elicited by pneumococcal carriage.

Many functions are attributed to IL-17A secreted from Th-17 cells [23]. It can enhance neutrophil recruitment and phagocytosis [18], increase antimicrobial peptide (beta defensin 2) production [24], iBALT formation [25], and enhance polymeric Immunoglobulin receptor expression on mucosal airway epithelial cells [26]. Human Th-17 cells persist for longer and are more resistant to apoptosis compared to Th-1 cells [27], making their increase an attractive goal for vaccinations relying on cellular immunity.

Nasopharyngeal pneumococcal carriage mediated alterations in the frequency and phenotype of pneumococcal-responding T-cell response(s) in the lung that could impact vaccination strategies to prevent acute lower respiratory tract infections or therapies designed to augment/modulate lung immunity. We have developed an Experimental Human Pneumococcal Carriage (EHPC) model to determine whether carriage could enhance cellular immunity to pneumococcus in the lung. We showed that carriage significantly increased lung and blood IL-17A^+^ CD4^+^ T-cell responses. Furthermore, rhIL-17A, dependent upon IL-17 receptor expression, can augment alveolar macrophage killing of pneumococci, to increase innate mucosal defences of the lung.

## Materials and Methods

### Volunteer Recruitment and Experimental Human Pneumococcal Carriage (EHPC) Model

Written informed consent was obtained from healthy adult volunteers to participate in an approved study at the Royal Liverpool and Broadgreen University Hospitals Trust. Approval was obtained from Liverpool Central [REC 11/NW/0011] and Sefton [08/H1001/52]) NHS Research Ethics Committees.

This work built on other EHPC development studies [28]. In contrast to our previous pneumococcal challenge study design [28] we omitted pre-challenge bronchoscopy with lavage from these cohorts to increase our colonisation success rate. Pneumococcal inoculation was done as published on-line [29]. Briefly, volunteers (cohort details in Table 1) were challenged with a single intra-nasal dose of either 23F (P833 strain a gift from Prof. JN Weiser, University of Pennsylvania) or 6B (BHN418 strain a gift from Prof. PW Hermans, University of Nijmegen) grown in vegitone broth (Oxoid). The inoculation was performed while the volunteer was seated comfortably in a semi-recumbent position. The head was tilted back slightly and 100 µl of the bacterial inoculum was dispensed, using a Gilson pipette (P100), across the nasal mucosa. Serial dilutions of the inoculum were plated onto blood agar (Oxoid) both before and after inoculation to confirm the dose (Table 1).

**Table 1:** Study volunteer and colonisation details.

Volunteer	Gender	Age	Pneumococcal Serotype	Dose (CFU per Naris/100 µl)	Time between inoculation and BAL/blood collection (d)	NW Colonisation Density (CFU/ml at 48 hr, 7 d or 14 d, respectively)			% IL-17A^+^ cells (% of total memory CD4^+^ T-cells in BAL)†
1	F	23	23F	44,167	55	N.D.			0.13
2	M	23	23F	44,167	44	N.D.			0.69
3	F	22	6B	19,500	56	N.D.			0.18
4	F	24	6B	49,500	21	1	174	362	2.67
5	M	29	6B	11,166	32	1	14	34	0.49
6	M	24	6B	11,166	30	13	0	0	1.7
7	F	22	6B	53,000	31	681	14,502	13	2.9
8	F	20	6B	82,000	31	173	265	292	0
9	F	22	6B	128,666	32	5,808	1,059	1,376	1.31
10	F	23	6B	92,333	28	1,214	1	573	6.99
11	F	20	6B	82,167	36	2	182	37	0.19
12	M	18	6B	136,667	25	114	1,805	1,795	N.D.
Total	8∶4 (F∶M)	22.5±2.7	10∶2 (6B∶23F)	66,617±45,637	35±10.6	N.D.			1.57±2.07
Controls	6∶3 (F∶M)	23±4.1	N/A	N/A	N/A	N/A			0.09±0.08

**N.D. – Not determined.**

**N/A – Not applicable.**

† **- Values given are background (media control) subtracted from cells stimulated with HK-6B.**

Intra-nasal colonisation was assessed in nasal washes collected 48 hours, 7 and 14 days later. Sterile isotonic saline (5 mls) was instilled into each naris with the subject seated at 45° to the horizontal. Saline was held in the nasopharynx for 5 seconds, following which the subjects were asked to tip their head gently forward to allow the saline to run out of the nose and be collected into specimen pots. Collected, pooled nasal washes were centrifuged at 3345 g for 10 minutes and the pellet was resuspended in 100 µl of Skim milk tryptone glucose glycerol (STGG) medium. An aliquot (25 µl) was plated onto Columbia horse blood agar (Oxoid) containing gentamicin (Sigma) and incubated at 37°C, 5% CO_2_. After 24 hours plates were inspected for the presence of draughtsman-like pneumococcal colonies. Isolated colonies were subsequently sub-cultured to confirm pneumococcal phenotype using Optochin sensitivity, bile solubility tests and for serotype confirmation, latex agglutination kits (Statens Serum Institute) were used. A further aliquot was used to perform a serial dilution (Miles and Misra) and 3×10 µl drops per dilution were dropped onto blood agar for colony counting to determine the carriage density (Table 1). Carriage density was calculated by obtaining the average CFU per 100 µl (of STGG) and dividing this value by the volume (ml) of nasal wash recovered to obtain CFU/ml of nasal wash. Volunteers with a pneumococcal positive nasal wash that was of the same serotype as the original inoculum were defined as having established carriage. These volunteers were subsequently selected for blood and BAL collection and subject data is summarised in Table 1. We also recruited 9 age-matched healthy adults (without pneumococcal colonisation) to act as controls and obtained BAL and blood samples (Table 1 and presented in [28]) for comparison in a cross-sectional study.

### PBMC and BAL Processing

PBMCs were processed by standard methods [28]. Briefly, PBMCs were seeded in 48-well tissue culture plates in RPMI 1640 media with 2 mM L-glutamine (both Sigma-Aldrich) and 10% human AB serum (complete media) lot 655272 (Invitrogen, UK), prior to stimulation.

BAL was obtained and processed as previously described [28]. BAL cells were plated out into standard 24-well tissue culture plates (Greiner, UK) to allow macrophages to adhere for 3 hours at 37°C, 5% CO_2_. BAL cells were also allowed to adhere to 96-well tissue culture plates (Greiner, UK) for an opsono-phagocytic assay, described below. Non-adherent cells were collected from 24-well tissue culture plates, washed and the pellet re-suspended in 1 ml of complete media in 48-well plates (Greiner, UK) and incubated at 37°C, 5% CO_2_.

### PBMC and BAL Stimulation

For Intracellular Cytokine Staining (ICS), PBMCs or BAL cells (containing 1–2×10^5^ lymphocytes per well) were stimulated *ex vivo* for 2 hours with influenza or one of the following pneumococcal antigen preparations: 1.0 µg/ml heat-killed 6B cells (HK-6B), 13 µg/ml (of which 4.2 µg/ml is pneumococcal protein) 6B culture supernatant (6B c/s), 13 µg/ml vegitone broth (‘vehicle’), 0.45 µg/ml of heat-inactivated influenza (Split Virion, Sanofi Pasteur, 2010/11 strains) or left untreated (‘NS’) [28]. After 2 hours, 1 µl of Brefeldin A (BD biosciences) was added and cells incubated for a further 16 hours before harvesting and staining for the presence of intracellular cytokines.

An equal number of BAL cells were also seeded in parallel and at an equal density to that described for ICS. These cells were stimulated for 20 hours with HK-6B, or left untreated as described above. Cells were harvested, pelleted and the supernatant removed and kept at −80°C for cytokine/protein measurements. There were no significant differences in the total number of cells, macrophages (mean [±SD] 8.5±5.5×10^5^ vs 8.4±7.5×10^5^) or lymphocytes (1.8±0.25×10^5^ vs 2.0±0.09×10^5^) per well between non-colonised and colonised groups, respectively.

### Flow Cytometry

Cells were harvested, stained and analysed as previously described [28]. We gated on viable, CD3^+^CD4^+^CD45RO^+^ T-cells (hereafter described as CD4^+^ memory T-cells) and identified individual TNF, IL-17A and/or IFNγ producing cells (or combinations thereof) following stimulation (Figure S1 in the online repository). Alveolar macrophage expression of IL-17RA and RC was determined as described elsewhere [30].

### Measurement of Secreted Proteins in Stimulated BAL Cell Supernatant

BAL cell culture supernatants (not treated with Brefeldin A) were analysed using a Th-1, Th-2, Th-17 Cytometric Bead Array (CBA) kit (Becton Dickinson, UK). IL-22 and Beta-defensin 2 (BD2) were measured, in duplicate, using an anti-human IL-22 ELISA (R and D Systems, UK) or an anti-human BD2 ELISA (Antigenix America Inc, USA), respectively. For the CBA, bead populations were acquired on a BD LSR 2 and fcs files analysed against the standard curve using FCAP version 1.0.1 (Soft Flow Inc. USA). For ELISAs optical density was measured at 450 nm using a Fluostar microplate reader (BMG Labtech, Germany) and corrected for background at 540 nm (IL-22) or corrected using empty wells (BD2). Standard curves were generated using linear regression fit (IL-22) or 4-parameter fit logistic regression (CBA and BD2) and had an r^2^ value greater than 0.97.

### Opsonophagocytic Killing Assay (OPKA)

An OPKA using pneumococci and human alveolar macrophages was performed with minor modifications [31]. Briefly, D39 Pneumococci (serotype 2) were opsonised in a 1∶16 dilution of intravenous immunoglobulin (IVIG, Gamunex, Talecris, USA) in Hanks and incubated at 37°C for 15 mins on a rotating platform. Opsonised D39 (20 µl), complement (10 µl) and either 20 µl of rhIL-17A (rhIL-17A, Biolegend 570502, reconstituted as described below) or vehicle control (HBSS [with Ca^2+^ Mg^2+^] containing 10% AB serum [lot 655272]) were added to 1×10^5^ adhered alveolar macrophages (multiplicity of infection of 1 pneumococcus :100 cells) in 30 µl of RPMI+10% FCS to give a total reaction volume of 80 µl in a 96-well flat bottom plate (Greiner, UK). Following 2 hours incubation at 37°C, 10 µl of reaction mixture was tilt plated, in triplicate, onto blood agar (Oxoid) and incubated at 37°C, 5%CO_2_ overnight. Colony forming units (CFUs) from cell supernatants were counted the following day.

### Statistical Analysis

Data with a normal distribution (tested by Shapiro Wilks) were compared with parametric tests. Data not following a normal distribution were compared with non-parametric tests. OPKA counts were assumed to follow a Poisson distribution. Changes in CFUs over the three rhIL-17A doses were examined using Poisson regression, with the corresponding vehicle counts included as covariates and with adjustment for clustering within participants. Flow cytometric data were analysed using FlowJo software version 7.6 (Treestar Oregon, USA). Graph and statistical analysis was performed using GraphPad prism version 5.0 (California, USA). Differences were considered significant if p≤0.05.

## Results

### Experimental Human Pneumococcal Carriage Can Be Achieved without Significant Side Effects

We recruited and inoculated 54 healthy young adult volunteers in a dose ranging study, with serotype 6B pneumococcus in which 20 volunteers established carriage (37%). In a 23F dose ranging cohort, 19 healthy adult volunteers were recruited and 2 established carriage (carriage positive 11%). In the 22 volunteers in whom we established carriage, 17 reported no symptoms, 4 reported mild upper respiratory flu-like symptoms and 1 reported abdominal pain and shortness of breath that resolved without therapy.

From the cohort of 22 volunteers with experimentally induced carriage, we were able to recruit 12 volunteers (average age 22.5 years) 36 days later (range 21–56 days) for BAL and blood sampling (6B n = 10; 23F n = 2, Table 1). These 12 carriage positive volunteers had been challenged with a mean dose of 66,617±45,637 CFUs (range 11,166–136,667 CFUs) per naris (Table 1). The proportion CD4^+^ memory T-cells positive for TNF, IL-17A or IFNγ were measured in BAL and blood and compared to controls without challenge (Table 1).

### Lung (BAL) and Blood Responses to Pneumococcus and Influenza in the Absence of Experimental Pneumococcal Colonisation

**Figure 1:**
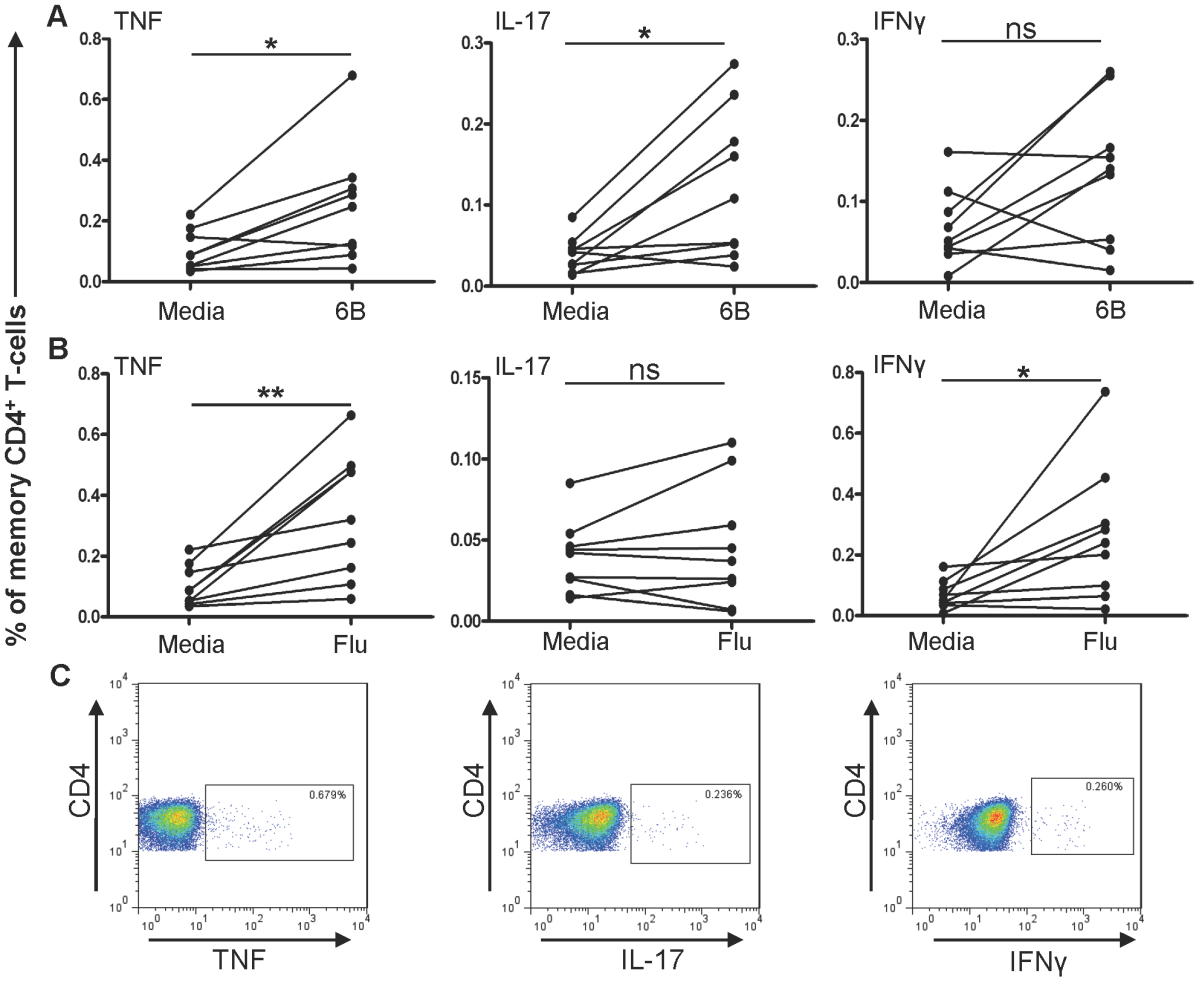
Pneumococcal-responding IL-17A^+^ and TNF^+^ CD4^+^ memory T-cells are present in BAL from non-colonised volunteers. **A** Heat-killed 6B pneumococci and **B** Influenza-antigen stimulated BAL cells from non-colonised volunteers (n = 9) were analysed for TNF, IL-17A and IFNγ expression using flow cytometry. TNF^+^, IL-17A^+^ or IFNγ^+^ cells were measured and recorded as a percentage of total CD4^+^ memory T-cells in BAL. **C** Representative flow cytometry dot-plots showing 6B stimulated CD4^+^ memory T-cells with the percentage of TNF^+^, IL-17A^+^ or IFNγ^+^ positive cells shown. * = p<0.05; ** = p<0.01 vs non-stimulated controls (media).

BAL cells and PBMCs from carriage negative volunteers were stimulated with pneumococcal antigens (HK-6B or 6B c/s) or influenza and cytokine (TNF, IL-17A or IFNγ) producing CD4^+^ memory T-cells were subsequently detected by ICS and flow cytometry. Pneumococcal-responding CD4^+^ memory T-cells were identified in BAL (Figure 1A and C and Figure S1 in the online repository) and PBMCs (Figure S2 in the online repository) in the absence of carriage. BAL CD4^+^ memory T-cells responding to heat-killed pneumococci (HK-6B) in the absence of carriage were TNF^+^ (0.25±0.19% vs media control 0.1±0.07% p = 0.02, paired T-test) and IL-17A^+^ (0.12±0.09% vs media control 0.04±0.02%, p = 0.01, paired T-test) but not IFNγ^+^, consistent with a Th-17 phenotype (Figure 1A). There was a positive correlation between TNF^+^ CD4^+^ memory T-cells and IL-17A^+^ CD4^+^ memory T-cells (Pearson r = 0.8; p = 0.009) in response to HK-6B. Similar observations were made when cells were stimulated *ex vivo* with concentrated pneumococcal culture supernatant (6B c/s) and compared to vegetone broth (vehicle) alone. We could detect pneumococcal-responding IL-17A^+^ (0.12±0.07 vs vehicle 0.06±0.05 p = 0.004) but not TNF^+^ or IFNγ^+^ CD4^+^ memory T-cells, again consistent with a Th-17 phenotype. In contrast, CD4^+^ memory T-cells responding to influenza stimulation, in the absence of carriage, were detectable in almost all BAL samples (Figure 1B and Figure S1 in the online repository) and these cells were TNF^+^ (0.33±0.21% vs media control 0.1±0.06%, p = 0.006, paired T-test) and IFNγ^+^ (0.27±0.22% vs media control 0.07±0.05%, p = 0.03, paired T-test) CD4^+^ memory T-cells, consistent with a Th-1 phenotype. IL-17A^+^ CD4^+^ memory T-cells were not detected in response to influenza (Figure 1B).

### Human Pneumococcal Carriage Elicits a Higher Frequency of IL-17A^+^ CD4^+^ Memory T-cells Compared to Volunteers without Carriage

#### BAL responses

**Figure 2:**
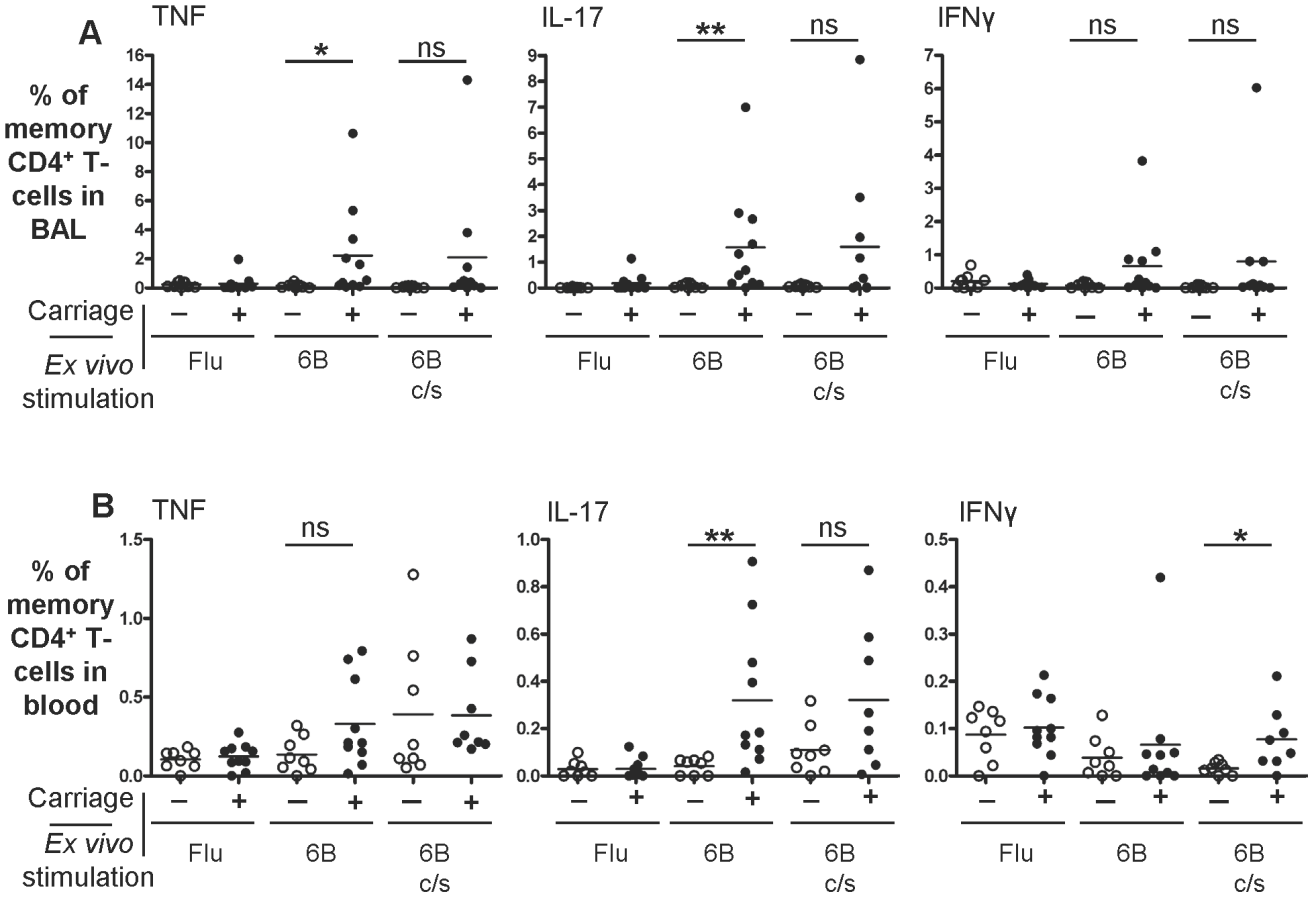
Pneumococcal carriage increases the percentage of pneumococcal-responding IL-17A^+^ CD4^+^ memory T-cells in BAL and blood. **A** BAL CD4^+^ memory T-cell and **B** Blood CD4^+^ memory T-cell responses from volunteers without (−, BAL n = 9, blood n = 8) or with (+, BAL n = 11, blood n = 10) experimental pneumococcal carriage (indicated on *x*-axis). Cells were stimulated *ex vivo* with or without (not shown) influenza or pneumococcal proteins as indicated. TNF^+^, IL-17A^+^ or IFNγ^+^ responses shown are background subtracted and measured as % of total CD4^+^ memory T-cells. Note the change in scale for the *y*-axis for each cytokine ns = not significant, * = *p*<0.05; ** = *p*<0.01 vs non-colonised volunteers.

**Figure 3:**
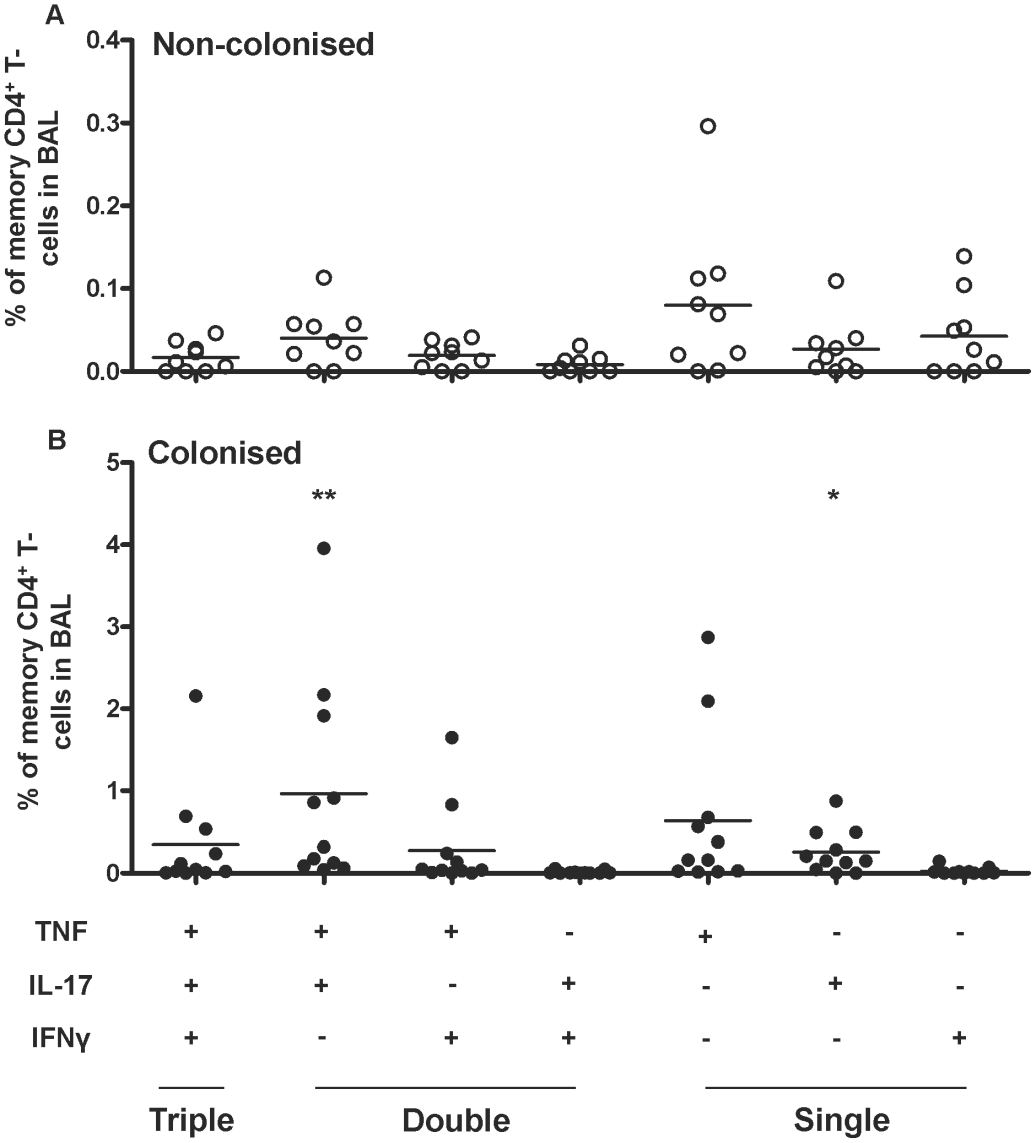
Pneumococcal carriage increases the percentage of IL-17A^+^/TNF^+^ CD4^+^ memory T-cells in BAL. Sub-population analysis of 6B stimulated BAL CD4^+^ memory T-cell responses from **A** non-colonised (n = 9) and **B** colonised volunteers (n = 11). The percentage of triple, double or single CD4^+^ memory T-cells producing either TNF, IL-17A and/or IFNγ (*x*-axis) are shown. Pneumococcal-responding double-producing (IL-17A^+^/TNF^+^) CD4^+^ memory T-cells constitute the dominant phenotype following pneumococcal colonisation. Responses shown are background subtracted. * = *p*<0.05; ** = *p*<0.01 vs non-colonised in **A**.

BAL differential cell counts from colonised and non-colonised volunteers were similar (Table 2). BAL cells collected from non-colonised and colonised groups and stimulated *ex vivo* with influenza antigen, showed a similar percentage of TNF^+^, IL-17A^+^ and IFNγ^+^ CD4^+^ memory T-cells (Figure 2A and Figure S2A and B in the online repository). In contrast, when BAL cells were stimulated with HK-6B, IL-17A^+^ CD4^+^ memory T-cells increased 17.4-fold in BAL following nasal carriage compared to volunteers without carriage. Following carriage 1.57±2.07% of total CD4^+^ memory T-cells were IL-17A^+^ compared to 0.09±0.08% without carriage (Figure 2A, p = 0.007). The IL-17A signal was derived mainly from IL-17A^+^ CD4^+^ memory T-cells that were also TNF^+^ (0.97±1.24% vs non-colonised 0.04±0.04%, p = 0.001) rather than single IL-17A^+^ cells (0.26±0.27% vs non-colonised 0.03±0.03%, p = 0.014), Figure 3. Stimulation of BAL cells from colonised volunteers with 6B c/s elicited higher frequencies of TNF^+^, IL-17A^+^ and IFNγ^+^ CD4^+^ memory T-cells but these responses were of borderline statistical significance (Figure 2A). Carriage did elicit cells with a Th-1 phenotype (IFNγ^+^CD4^+^ memory T-cells) in some volunteers but overall these findings were inconsistent and thus we could not demonstrate statistical significance. The proportion of IL-17A^+^ CD4^+^ memory T-cells in BAL did not correlate with the density of carriage (shown in Table 1).

**Table 2:** Cellular profile of BAL collected from the pneumococcal non-colonised and colonised groups.

	Non-Colonised	Colonised
Volunteers	*n* = 9	*n* = 12
Volume returned (ml)	116±22	122±29
Total cells (×10^6^/ml)	0.129±0.1	0.09±0.05
Macrophages (%)	93.4±3	93.7±4
Lymphocyte (%)	5.0±2	4.9±3
Neutrophil (%)	1.6±1.6	1.3±1.2

#### Blood responses

PBMCs from pneumococcal colonised (n = 10) and non-colonised (n = 8) adults stimulated *ex vivo* with influenza antigen elicited similar frequencies of TNF^+^, IL-17A^+^ and IFNγ^+^ CD4^+^ memory T-cells (Figure 2B). In contrast, HK-6B stimulated PBMCs from colonised volunteers showed a significantly greater percentage of IL-17A^+^ but not TNF^+^ or IFNγ^+^ CD4^+^ memory T-cells, corresponding to an 8-fold increase, compared to non-colonised volunteers (0.32±0.3% vs non-colonised 0.04±0.03%, p = 0.003). PBMCs from colonised volunteers stimulated with 6B c/s showed a greater percentage of IL-17A^+^ and IFNγ^+^ (0.08±0.07 vs non-colonised 0.02±0.01, p = 0.02) but not TNF^+^ CD4^+^ memory T-cells compared to non-colonised volunteers.

### BAL Cells Stimulated with Pneumococci Elicit High Levels of IL-17A but Not IFNγ

**Figure 4:**
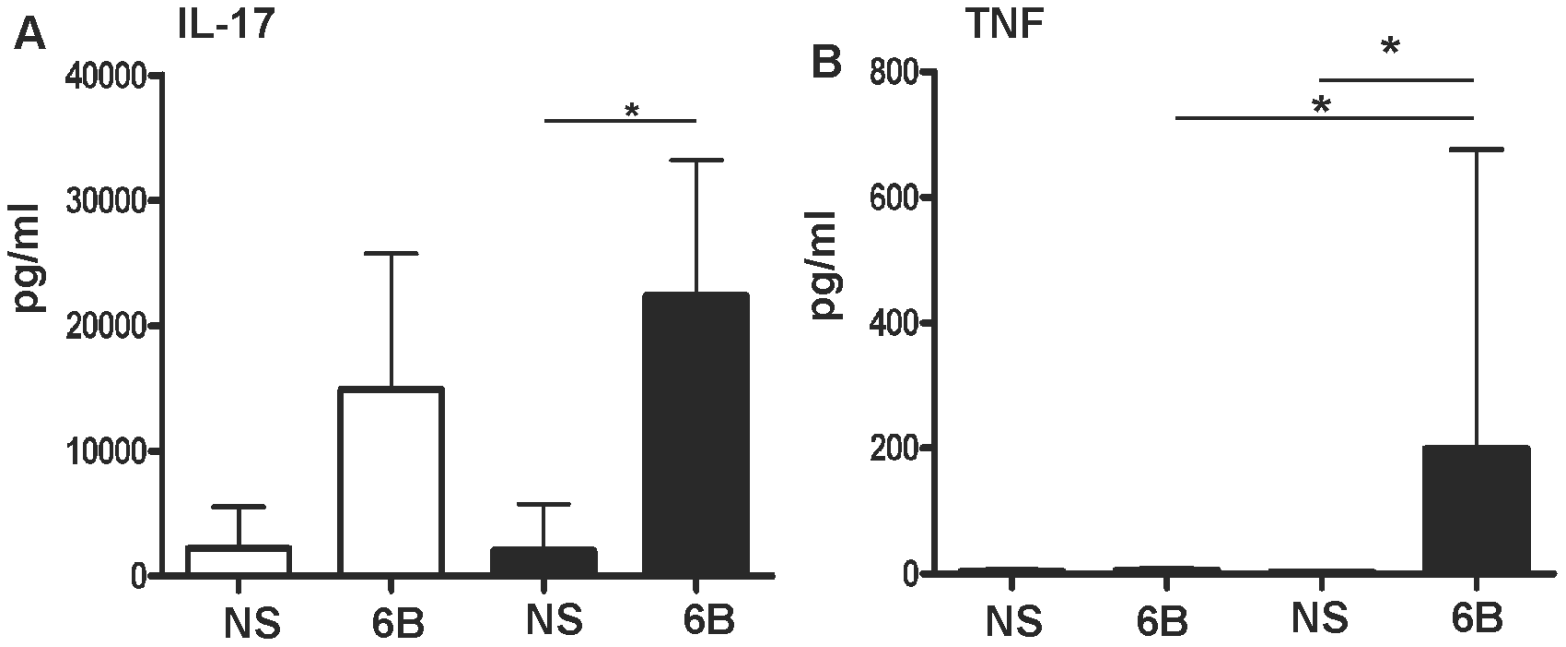
BAL cells stimulated with pneumococci *ex vivo* elicit large quantities of IL-17A. BAL cells from non-colonised (open bars, n = 6) or colonised (closed bars, n = 7, 1x 23F, 6x6B colonised) volunteers were stimulated with pneumococci (6B) or left untreated (NS) for 20 hours. Following stimulation **A** IL-17A and **B** TNF were measured in pg/ml (mean±SD) in cell culture supernatant by ELISA. * = *p*<0.05.

To corroborate our flow cytometry findings we stimulated BAL cells from colonised and non-colonised volunteers with HK-6B or left them untreated and measured secreted IL-17A (Figure 4A), TNF (Figure 4B), IL-2, IL-4, IL-6, IL-10, IL-22 and IFNγ (all Figure S4 in the online repository) by ELISA. Large quantities of IL-17A were detected in the culture supernatant of HK-6B stimulated BAL cells from non-colonised volunteers (stimulated mean±SD 14,907±10,843 vs non-stimulated 2,233±3,298 pg/ml, p = 0.06, Figure 4A). BAL cells from colonised volunteers and stimulated with HK-6B elicited significantly greater quantities of IL-17A protein compared to non-stimulated cultures (stimulated 22,393±10,830 vs non-stimulated 2,002±3,738 pg/ml, p = 0.03). HK-6B stimulated BAL cells from colonised volunteers produced 50% more IL-17A protein than HK-6B stimulated BAL cells from non-colonised volunteers but this difference was not statistically significant. IL-17A production did not correlate with the frequency of pneumococcal-responding IL-17A^+^ CD4^+^ T-cells in BAL detected by flow cytometry (r = 0.13, p = 0.68). IL-17A production did correlate with the number of alveolar macrophages per well (r = 0.59, p = 0.03) indicative of an alternative source of IL-17A, other than CD4^+^ memory T-cells, in BAL.

Comparisons between colonised and non-colonised groups, following pneumococcal stimulation, revealed no significant differences with the exception of TNF (198.9±476.8 vs non-colonised 5.8±3, p = 0.05 Mann-Whitney, Figure 4B). When corrected for background (by subtracting data from non-stimulated cells) the significance of this observation increased (TNF 195.7±476.5 vs non-colonised 2.67±2.45, p = 0.026 Mann Whitney).

We then hypothesised that the presence of IL-17A (and TNF) in stimulated culture supernatant would in turn elicit alveolar macrophage secretion of constitutively expressed BD2 protein [32] after 20 hrs but this was below the limit of detection in all samples (data not shown).

### Human Alveolar Macrophages Express Functional IL-17A Receptors and rhIL-17A Stimulation Increases Uptake of Pneumococci

**Figure 5:**
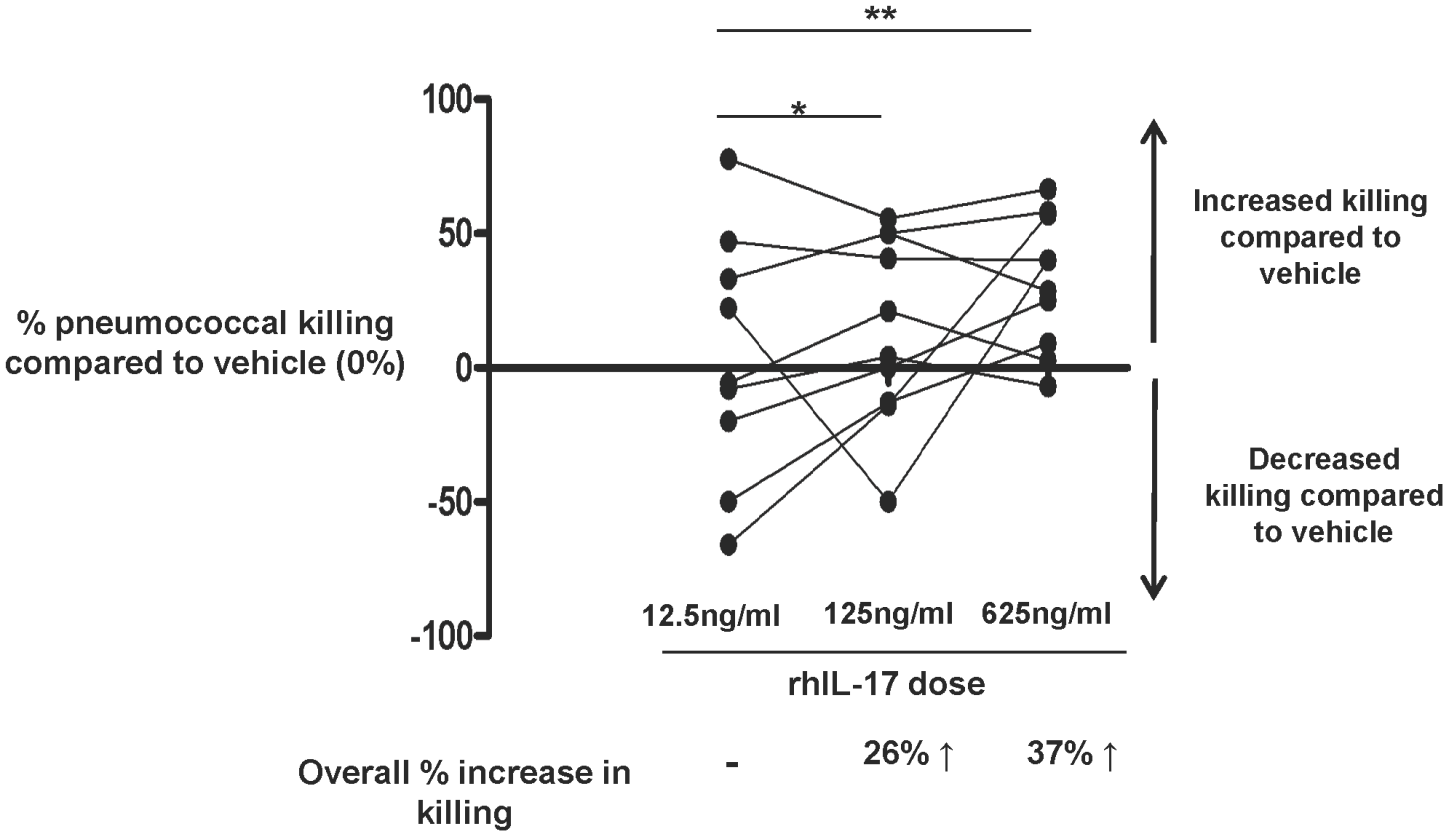
rhIL-17A stimulated alveolar macrophages show increased killing of opsonised pneumococci. Human alveolar macrophages from non-colonised volunteers were exposed to opsonised D39 pneumococci in the presence of rhIL-17A or a vehicle control (v/v). Percentage changes in CFU following rhIL-17A (*y*-axis) treatment are shown as increase or decrease relative to vehicle, which was set at 0% for comparison (*x*-axis). * = *p*<0.05, ** = *p*<0.01 vs 12.5 ng/ml of rhIL-17A.

Human alveolar macrophages expressed both IL-17 RA (7438±1646 mean channel units, n = 5) and RC (3551±2426 mean channel units n = 6) sub-units consistent with our previous observations [30]. We thus used rhIL-17A and a modified OPKA assay to mimic CD4 T cell action and our hypothesis was that rhIL-17A could enhance the anti-pneumococcal response (independent of serotype) of human alveolar macrophages. To calculate a percentage increase or decrease compared to vehicle treated cells, CFU averages from each rhIL-17A dose and respective control were divided to obtain a ratio (Figure 5 and raw data in Table 3). We showed a dose dependent increase in macrophage uptake of pneumococci using 12.5 ng/ml, 125 ng/ml or 625 ng/ml concentrations of rhIL-17A (Figure 5 and Table 3).

**Table 3:** CFU recovered in BAL cell culture supernatant following OPKA.

IL-17A (ng/ml)	Mean (95% confidence interval)			Ratio of IL-17A adjusted CFU/IL-17 adjusted CFU at 12.5 ng/ml dose (95% confidence interval) [p]
	Control	IL-17A unadjusted	IL-17A adjusted†	
**12.5**	13.5 (8.8–18.2)	13.8 (7.2–20.4)	14.2 (10.7–17.6)	—
**125**	16.8 (11.2–22.5)	13.7 (8.2–19.3)	10.5 (8.0–12.9)	0.739 (0.582–0.939) [p = 0.013]
**625**	16.8 (10.5–23.1)	12.4 (6.4–18.3)	8.9 (6.5–11.3)	0.629 (0.458–0.862) [p = 0.004]

† Adjusted for clustering within participants.

Macrophage uptake of pneumococci was increased 26% in the presence of 125 ng/ml of rhIL-17A, compared to the 12.5 ng/ml dose (12.5 ng/ml Control: 14.2 vs rhIL-17A stimulated 10.5 CFU p = 0.013). Increasing the rhIL-17A dose to 625 ng/ml further increased pneumococcal uptake to 37% (12.5 ng/ml Control: 14.2 vs rhIL-17A stimulated 8.9 CFU p = 0.004) (Figure 5 and Table 3).

We correlated the OPKA data described above with IL-17 receptor RA and RC mean fluorescence intensity on BAL alveolar macrophages from a sub-set of the same volunteers (n = 6) to determine whether this response was mediated by the IL-17 receptor. Our hypothesis was that increased mean receptor expression positively correlates with increased percentage killing compared to vehicle at the 125 ng/ml dose. There were no significant correlations between OPKA data and expression of RA or combined expression of RA and RC. Contrary to our hypothesis, however, mean expression of RC (3551±2426), negatively correlated with killing (Spearman r = −0.9, p = 0.017).

## Discussion

We have shown that pneumococcal-responding IL-17A^+^ CD4^+^ memory T-cells are present at very low frequency in the healthy adult lung in the absence of carriage. Further, using a novel experimental human pneumococcal carriage (EHPC) model and post carriage BAL collection an episode of pneumococcal carriage resulted in a 17.4-fold increase and 8-fold increase in the percentage of IL-17A^+^ CD4^+^ memory T-cells in BAL and blood, respectively, compared to non-colonised volunteers. Using human alveolar macrophages as effectors we showed that rhIL-17A increased *in vitro* killing of *S. pneumoniae* in an opsonophagocytic killing assay. These are the first data of which we are aware to describe the relation of nasal carriage of a pathogenic organism and lung IL-17A responses in humans and together support a role for effector IL-17A^+^ CD4^+^ memory T-cell responses in the defence of the lung against pneumococcal infection in adults.

The two major strengths of this study are that we have described human CD4^+^ T-cell responses in the relevant mucosal site and after a defined period of nasal colonisation. Other investigators have identified and described pneumococcal-responding human CD4^+^ T-cells in blood [13], [19], [20], [33], [34] and upper respiratory tract mucosal tissue [13], [20], [35] but the initiation and duration of carriage was unknown. The sharp increase in cellular response seen immediately following an episode of carriage in this study, and not seen in similar volunteers challenged with live bacteria but without carriage [28], strongly supports a lung immunising role of carriage in adults. Although a study of subjects at high risk of pneumococcal disease (children, elderly) would be immunologically more relevant, it would clearly be ethically unacceptable in the context of human pathogen challenge.

Our data contrast with decreases in antigen-specific responses observed in blood in pneumococcal carriers in UK children [13] or in endemic areas such as the Gambia [33] and in UK patients with pneumonia [14], probably due to mucosal sequestration. Our data concur, however, with increased IL-17A responses in other studies [17] from an area with a high prevalence of pneumococcal carriage and disease (i.e. Bangladesh) compared to Swedish cohorts. The difference between our study and others may be due to differences in the timing of sample collection relative to exposure.

Human pneumococcal-responding IL-17A responses have been demonstrated previously in peripheral blood [17], [18], [33] and in adenoidal mono-nuclear cells [20], [35] but in our study we have examined the mucosal compartment where pneumonia becomes established – the lung, which has not been examined before. In a healthy adult population we showed, using flow cytometry, that pneumococcal carriage elicits high frequencies of IL-17A^+^ and TNF^+^ but not IFNγ^+^ cells, within 5 weeks of colonisation. We have used an extensive 7-colour panel that includes CD3^+^ and CD4^+^ antibodies that together with IL-17^+^ detection ensures that it is highly likely that the responses we have identified in this manuscript are derived from putative Th-17 cells rather than innate sources. The IL-17A dominant responses in BAL and blood contrast with studies that described higher IFNγ and lower IL-17A responses in blood [19], [33] and tonsil [20] from healthy and HIV affected [19] adults in Malawi and Gambia. There are multiple factors, including the tissue site examined, burden of disease and cellular plasticity [36] that may account for higher IFNγ in these studies and these differences between geographical areas of high and low pneumococcal carriage warrant further attention. It is likely, however, that both IL-17A and IFNγ from T-cell effector cells as well as T-regulatory cell populations play important but different roles in protecting the lung against the pneumococcus and pneunomococcal induced pathology [3], [37]–[40]. Furthermore, we identified increased IL-22 levels in some volunteers that were independent of the IL-17 response suggesting a separate source of IL-22 (possibly Th-22 cells), the diverse functions of which include maintenance of epithelial integrity [41] and remodelling [42]. Murine models of airway inflammation have shown that IL-22 can be pro-inflammatory (and thus pathological) in the presence of IL-17A but in the absence of IL-17A can be anti-inflammatory/tissue protective [43]. Determining the correct “balance” of Th-17, 22 and T-regulatory cells elicited following vaccination may be important for generating adaptive anti-pneumococcal responses that promote resolution and clearance and reduce immunopathology.

We have also measured the cytokine response from lung cells stimulated *ex vivo* with pneumococcus and shown that pneumococcal stimulated BAL cells (from non-colonised and colonised volunteers) produce IL-17A in quantities far greater than described in other studies using blood [17]–[19] or lymphoid tissue [20], [35]. The response from colonised volunteers was 50% greater compared to the non-colonised group who also had high levels of IL-17A following stimulation that is likely to be derived from non-Th-17 sources. This difference may be of relevance *in vivo*, however, since TNF [44], which we have shown to be significantly different between colonised and non-colonised groups using flow cytometry (Figure 2A) and ELISA (Figure 4B), and IL-22 [45] can both synergise with IL-17A to enhance epithelial derived CXC chemokine production, important for the recruitment and activation of neutrophils to the airway. It has been shown that murine [46] and human [47] alveolar macrophages can also produce IL-17A utilising a TLR-2 dependent mechanism [46] and this may have contributed to the IL-17A signal detected by our ELISA in both groups. An important role for the alveolar macrophage in the early hours of pneumococcal infection has been highlighted previously in murine models [48] and IL-17A from innate sources are likely to be involved [49].

In this study we showed a significant increase in pneumococcal killing by macrophages when exposed to 125 ng/ml of rhIL-17A, a concentration that is in line with previous publications showing an effect of IL-17 in this dose range [18], [45], [50]–[52]. This is also consistent with the study by Lu *et al.* [18] who showed that human neutrophils exposed to 100 ng/ml of rhIL-17A showed significantly increased pneumococcal killing. Our data are also in line with those of Higgins *et al.* [53] who showed that treatment of murine peritoneal macrophages with 2.5–50 ng/ml of rmIL-17A significantly enhanced killing of *Bordetella pertussis*. IL-17 also acts as a recruitment and survival factor for monocytes and macrophages [54], respectively, thus promoting macrophage-Th-17 interaction in the small volume of airway lining fluid [55].

Both RA and RC subunits are required for human IL-17A signalling with combined surface receptor density of RA and RC determining the magnitude of the response [56] but we did not find any positive correlations between our OPKA data and receptor expression. In contrast to our hypothesis, we observed a negative correlation between IL-17RC (but not RA) expression and macrophage killing activity at the 125 ng/ml dose. The modulation of killing by RC supports our observations of an IL-17-dependent effect in our assay system rather than a contaminant. Furthermore the negative correlation between IL-17RC (but not RA) expression and macrophage killing suggests that killing may be mediated by a different IL-17RA heterodimer other than RA:RC. RC may thus play a regulatory role in this process, separate from its pro-inflammatory role within the RA:RC dimer, fine tuning the phagocytic potential of alveolar macrophages and thus susceptibility to infection. There is evidence that IL-17 receptors play regulatory roles during the inflammatory response [30], [52]. Recent observations have shown that RD expression intensity can differentially regulate p38 mitogen-activated protein kinase and nuclear factor-kappa B pathways and more importantly the control of lung neutrophil recruitment in a CXCL2 dependent manner [52]. Evidence provided here and elsewhere thus suggests that the role of IL-17 receptors is more complex than initially appreciated and may differ depending on the context. The role of IL-17 receptors, other than the classical IL-17RA:RC heterodimer, on alveolar macrophage function in health and disease remains to be clarified and may determine the overall protective effect of Th-17 cells.

Taken as a whole, these results have important implications for vaccine design against pneumonia. First, they show that human nasal carriage can boost innate (alveolar macrophage function) and adaptive (TNF^+^IL-17A^+^ CD4^+^ memory T-cells) cellular lung immunity that may protect the lung from pneumococcal challenge and the establishment of infection in health, without significant recruitment of neutrophils. When these and other protective immunological mechanisms are compromised or the bacterial load overwhelms innate defence mechanisms the responses described in our study may synergise to enhance neutrophil mediated recruitment into the airspace. Second, we have begun to define the phenotypic and kinetic cellular responses elicited by pneumococcal carriage – a natural immunising event, thus providing a bench mark for vaccines that seek to protect against pneumonia.

## Supporting Information

Figure S1 **Flow Cytometry gating strategy to detect cytokine positive CD4^+^ memory T cells in BAL.** **A** BAL Lymphocytes were identified on FSC and SSC analysis. **B** Gated cells were measured for viability (Vivid negative) and CD3 expression. **C** Gated cells from **B** (viable CD3^+^ T-cells) were then measured for CD3 and CD45RO expression to identify memory (CD45RO^+^) CD3^+^ T-cells. **D** Viable CD3^+^ memory T cells were gated onto a bivariate dot plot measuring CD3 and CD4 to identify BAL CD4^+^ T-cells (black circle). CD4^+^ T-cell events from **E** were gated onto a bivariate dot plot with CD3 expression on the y-axis and either **E** and **H** TNF (AF_488_) or **F** and **I** IL-17A (PE) or **G** and **J** IFNγ (AF_700_) on the x-axis. Shown are CD4+ T-cell responses when cultured in **E**, **F** and **G** media alone or following stimulation with **H**, **I** and **J** influenza-antigen. Cytokine positive events were identified as shown in **E–J** (boxed areas) and reported as a percentage of total CD4^+^ memory T-cells for each condition.
(TIFF)

Figure S2 **Pneumococcal-responding IL-17A^+^ and TNF^+^ CD4^+^ memory T-cells are present in blood from non-colonised volunteers.** **A** 6B pneumococcal culture supernatant **B** heat-killed 6B pneumococci and **C** Influenza stimulated PBMCs, from non-colonised volunteers (n = 8), analysed for TNF, IL-17A and IFNγ expression as a proportion of CD4^+^ memory T-cells. We detected a low frequency of TNF^+^ and IL-17A^+^ CD4^+^ memory T-cells in response to pneumococcal stimulation compared to vehicle control treated cells. * = *p*<0.05.
(TIFF)

Figure S3 **Pneumococcal Carriage does not significantly alter the influenza CD4^+^ T-cell response in BAL.** BAL cells from **A** non-colonised (n = 9) and **B** colonised volunteers (n = 11) were stimulated with influenza. CD4^+^ memory T-cell expression of TNF, IL-17A and/or IFNγ, as indicated in legend, was measured and recorded as a percentage. Responses shown are background subtracted. In non-colonised volunteers BAL CD4 T cell responses constitute mostly double (TNF/IFNγ) and single (TNF or IFNγ) producing T cells. From pneumococcal colonised volunteers, influenza specific responses are similar.
(TIFF)

Figure S4 **BAL cells stimulated with pneumococci elicit IL-2, IL-4, IL-6 IL-10, IL-22 but not IFNγ.** BAL cells from non-colonised (open bars, n = 6) or colonised volunteers (closed bars, n = 7) were left untreated (NS) or stimulated with pneumococci (6B). Cell culture supernatants from non-colonised and colonised volunteers were collected after 20 hours and measured for the presence of IFNγ, IL-2, IL-4, IL-6, IL-10 and IL-22 in pg/ml as shown. * = *p*<0.05.
(TIFF)
