# Supplementary figures and images for: Experimental Human Pneumococcal Carriage Augments IL-17A-dependent T-cell Defence of the Lung
Source: PLoS Pathog. 2013 Mar 28;9(3):e1003274. doi: 10.1371/journal.ppat.1003274 (PMC3610738; doi:10.1371/journal.ppat.1003274)

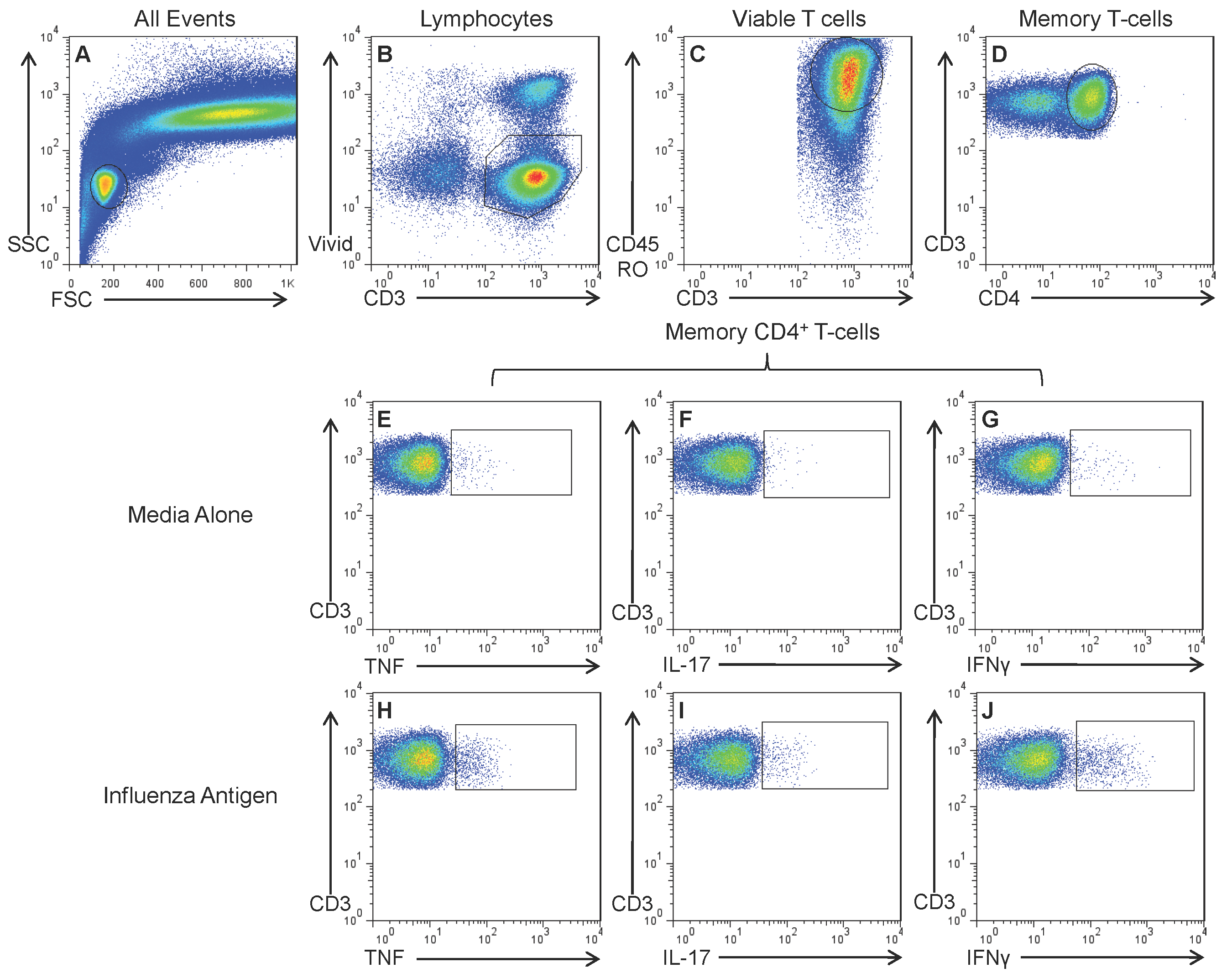

Supplement: Figure S1 — Flow Cytometry gating strategy to detect cytokine positive CD4+ memory T cells in BAL. A BAL Lymphocytes were identified on FSC and SSC analysis. B Gated cells were measured for viability (Vivid negative) and CD3 expression. C Gated cells from B (viable CD3+ T-cells) were then measured for CD3 and CD45RO expression to identify memory (CD45RO+) CD3+ T-cells. D Viable CD3+ memory T cells were gated onto a bivariate dot plot measuring CD3 and CD4 to identify BAL CD4+ T-cells (black circle). CD4+ T-cell events from E were gated onto a bivariate dot plot with CD3 expression on the y-axis and either E and H TNF (AF488) or F and I IL-17A (PE) or G and J IFNγ (AF700) on the x-axis. Shown are CD4+ T-cell responses when cultured in E, F and G media alone or following stimulation with H, I and J influenza-antigen. Cytokine positive events were identified as shown in E–J (boxed areas) and reported as a percentage of total CD4+ memory T-cells for each condition. (TIFF) [file ppat.1003274.s001.tiff]

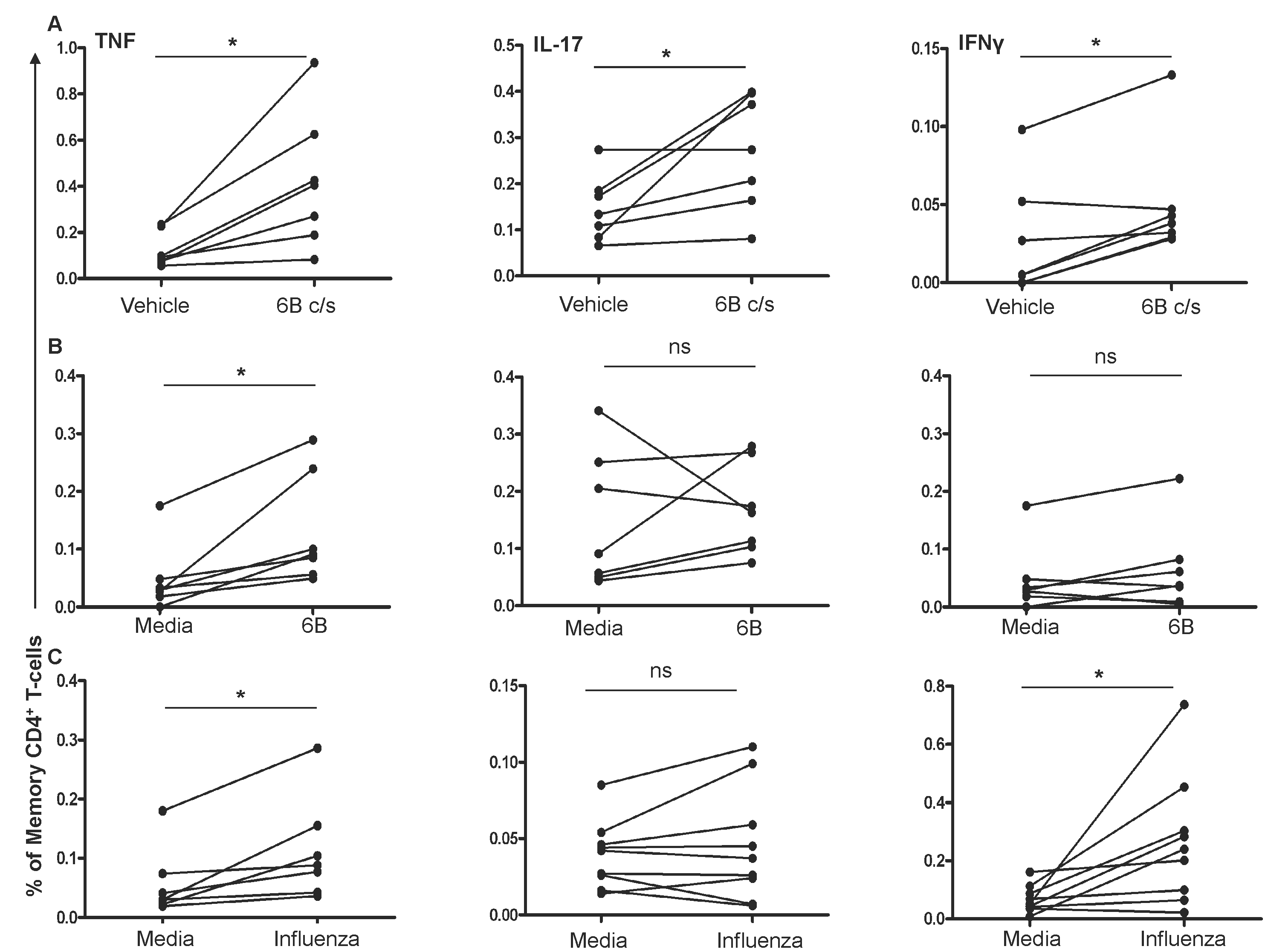

Supplement: Figure S2 — Pneumococcal-responding IL-17A+ and TNF+ CD4+ memory T-cells are present in blood from non-colonised volunteers. A 6B pneumococcal culture supernatant B heat-killed 6B pneumococci and C Influenza stimulated PBMCs, from non-colonised volunteers (n = 8), analysed for TNF, IL-17A and IFNγ expression as a proportion of CD4+ memory T-cells. We detected a low frequency of TNF+ and IL-17A+ CD4+ memory T-cells in response to pneumococcal stimulation compared to vehicle control treated cells. * = p<0.05. (TIFF) [file ppat.1003274.s002.tiff]

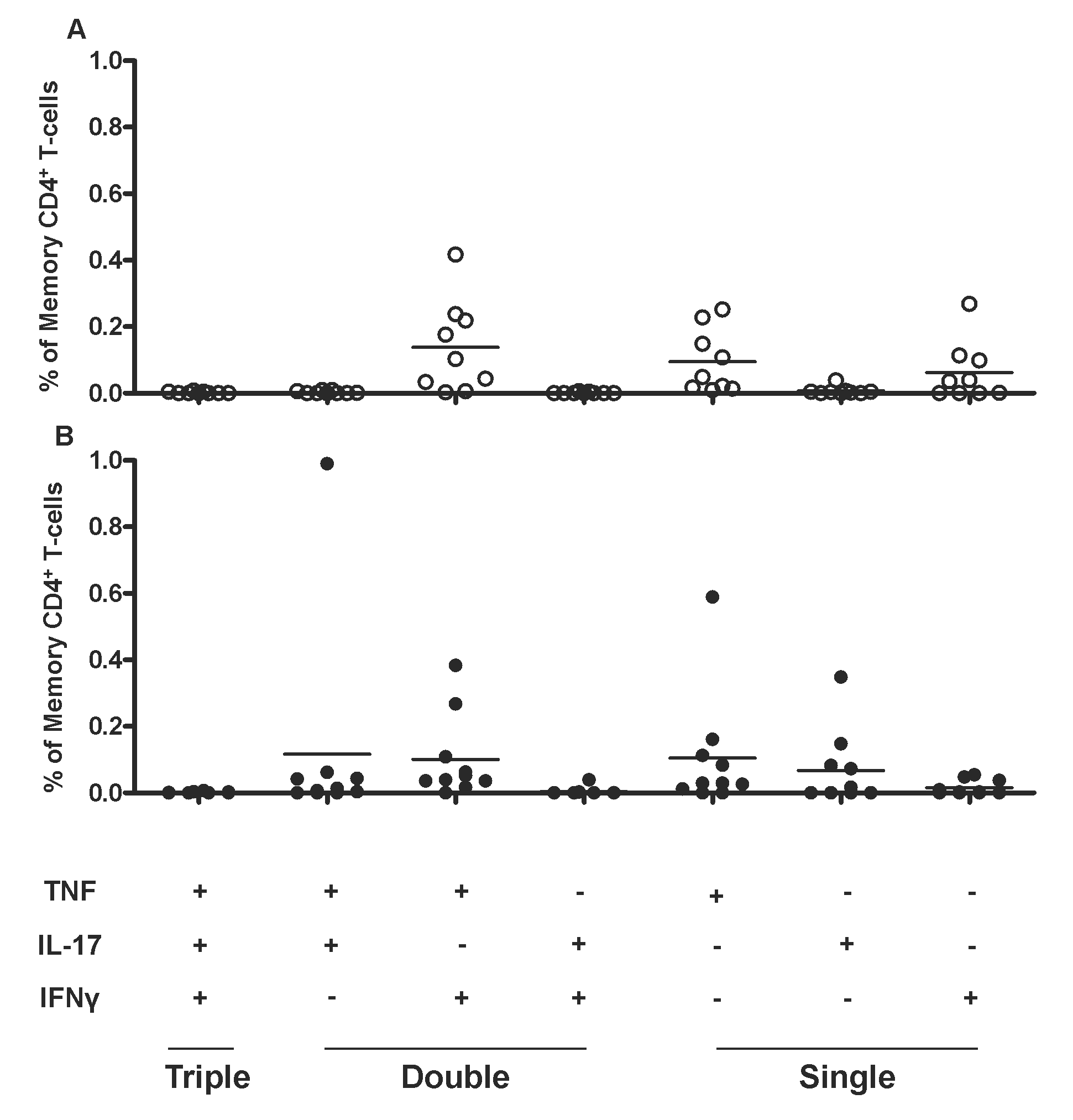

Supplement: Figure S3 — Pneumococcal Carriage does not significantly alter the influenza CD4+ T-cell response in BAL. BAL cells from A non-colonised (n = 9) and B colonised volunteers (n = 11) were stimulated with influenza. CD4+ memory T-cell expression of TNF, IL-17A and/or IFNγ, as indicated in legend, was measured and recorded as a percentage. Responses shown are background subtracted. In non-colonised volunteers BAL CD4 T cell responses constitute mostly double (TNF/IFNγ) and single (TNF or IFNγ) producing T cells. From pneumococcal colonised volunteers, influenza specific responses are similar. (TIFF) [file ppat.1003274.s003.tiff]

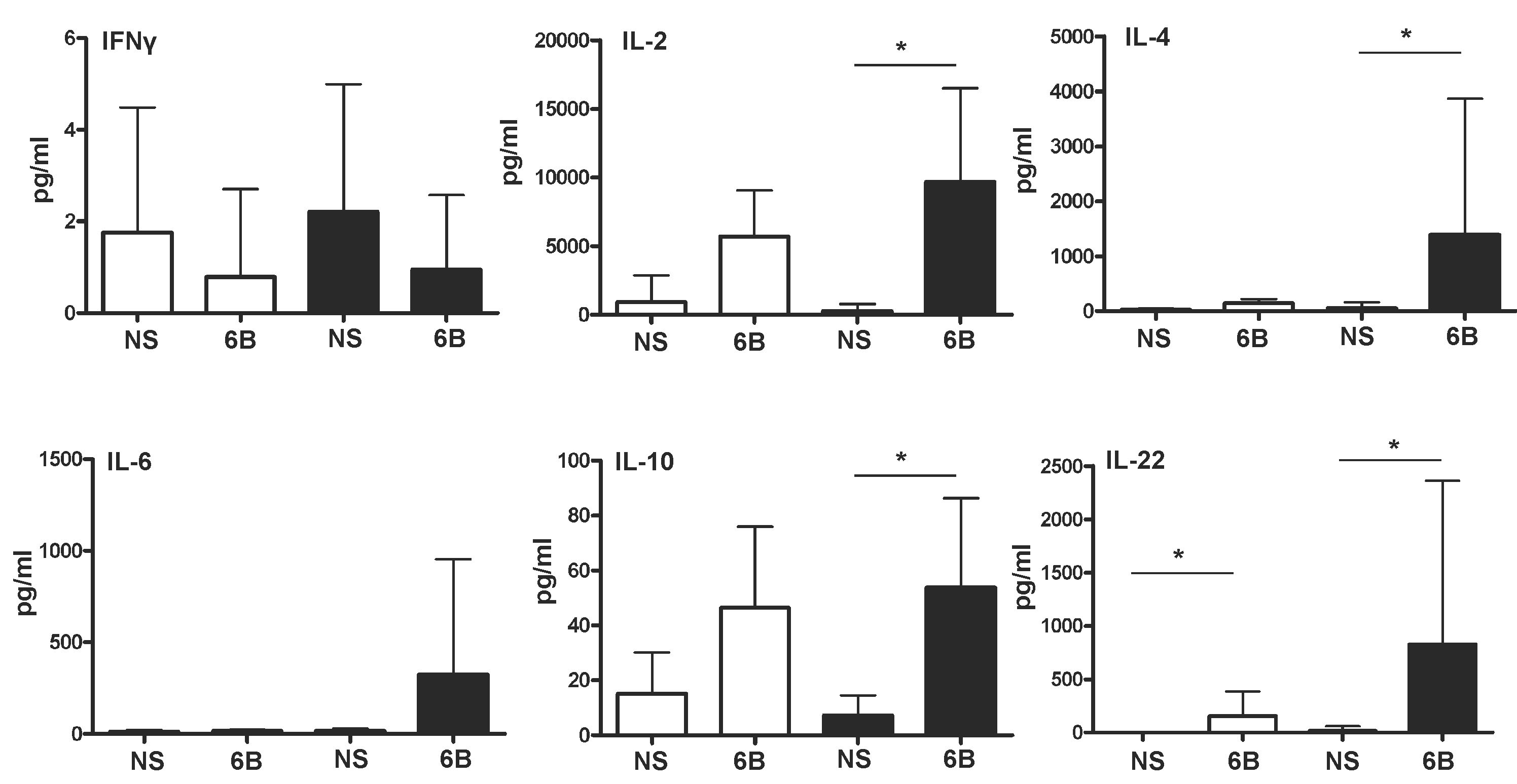

Supplement: Figure S4 — BAL cells stimulated with pneumococci elicit IL-2, IL-4, IL-6 IL-10, IL-22 but not IFNγ. BAL cells from non-colonised (open bars, n = 6) or colonised volunteers (closed bars, n = 7) were left untreated (NS) or stimulated with pneumococci (6B). Cell culture supernatants from non-colonised and colonised volunteers were collected after 20 hours and measured for the presence of IFNγ, IL-2, IL-4, IL-6, IL-10 and IL-22 in pg/ml as shown. * = p<0.05. (TIFF) [file ppat.1003274.s004.tiff]
